# Development and Characterization of Dual-Loaded Niosomal Ion-Sensitive In Situ Gel for Ocular Delivery

**DOI:** 10.3390/gels10120816

**Published:** 2024-12-11

**Authors:** Viliana Gugleva, Rositsa Mihaylova, Katya Kamenova, Dimitrina Zheleva-Dimitrova, Denitsa Stefanova, Virginia Tzankova, Maya Margaritova Zaharieva, Hristo Najdenski, Aleksander Forys, Barbara Trzebicka, Petar D. Petrov, Denitsa Momekova

**Affiliations:** 1 Department of Pharmaceutical Technologies, Faculty of Pharmacy, Medical University of Varna, 84 Tsar Osvoboditel Str., 9000 Varna, Bulgaria; viliana.gugleva@mu-varna.bg; 2 Department of Pharmacology, Pharmacotherapy and Toxicology, Faculty of Pharmacy, Medical University of Sofia, 2 Dunav Str., 1000 Sofia, Bulgaria; rmihaylova@pharmfac.mu-sofia.bg (R.M.); denitsa.stefanova@pharmfac.mu-sofia.bg (D.S.); vtzankova@pharmfac.mu-sofia.bg (V.T.); 3 Institute of Polymers, Bulgarian Academy of Sciences, bl.103 Akad. G. Bonchev St., 1113 Sofia, Bulgaria; kkamenova@polymer.bas.bg (K.K.); ppetrov@polymer.bas.bg (P.D.P.); 4 Department of Pharmacognosy, Faculty of Pharmacy, Medical University of Sofia, 2 Dunav Str., 1000 Sofia, Bulgaria; dzheleva@pharmfac.mu-sofia.bg; 5 Department of Infectious Microbiology, The Stephan Angeloff Institute of Microbiology, Bulgarian Academy of Sciences, 26 Acad. G. Bonchev Str., 1113 Sofia, Bulgaria; zaharieva26@yahoo.com (M.M.Z.); hnajdenski@gmail.com (H.N.); 6 Centre of Polymer and Carbon Materials, Polish Academy of Sciences, ul. M. Curie-Skłodowskiej 34, 41-819 Zabrze, Poland; aforys@cmpw-pan.pl (A.F.); btrzebicka@cmpw-pan.pl (B.T.); 7 Department of Pharmaceutical Technology and Biopharmaceutics, Faculty of Pharmacy, Medical University of Sofia, 2 Dunav Str., 1000 Sofia, Bulgaria

**Keywords:** cannabidiol, epigallocatechin-3-gallate, gellan gum, HaCaT cells, ocular delivery, rheological studies, sol gel transition

## Abstract

The study investigates the development and characterization of dual-loaded niosomes incorporated into ion-sensitive in situ gel as a potential drug delivery platform for ophthalmic application. Cannabidiol (CBD) and epigallocatechin-3-gallate (EGCG) simultaneously loaded niosomes were prepared via the thin film hydration (TFH) method followed by pulsatile sonication and were subjected to comprehensive physicochemical evaluation. The optimal composition was included in a gellan gum-based in situ gel, and the antimicrobial activity, in vitro toxicity in a suitable corneal epithelial model (HaCaT cell line), and antioxidant potential of the hybrid system were further assessed. Dual-loaded niosomes based on Span 60, Tween 60, and cholesterol (3.5:3.5:3 mol/mol) were characterized by appropriate size (250 nm), high entrapment efficiency values for both compounds (85% for CBD and 50% for EGCG) and sustained release profiles. The developed hybrid in situ gel exhibited suitable rheological characteristics to enhance the residence time on the ocular surface. The conducted microbiological studies reveal superior inhibition of methicillin-resistant *Staphylococcus aureus* (MRSA) adhesion by means of the niosomal in situ gel compared to the blank gel and untreated control. Regarding the antioxidant potential, the dual loading of CBD and EGCG in niosomes enhances their protective properties, and the inclusion of niosomes in gel form preserves these effects. The obtained outcomes indicate the developed niosomal in situ gel as a promising drug delivery platform in ophthalmology.

## 1. Introduction

Ocular drug delivery via conventional dosage forms, such as eye drops, is often associated with insufficient bioavailability (less than 5%), which may be attributed to two main factors—the anatomical structure of the human eye and associated static and dynamic barriers—preventing the instillation of foreign substances, as well as the liquid nature and large volume of the applied drop, which determines the observed short residence time and fast elimination [1,2]. Since the anatomical and physiological features of the human eye are constant and, therefore, can not be tuned, research efforts are focused on the optimization of the technological characteristics of conventional dosage forms and the elaboration of nanoscale carriers and novel drug delivery platforms. Commonly used approaches for improving the limitations of eye drops include the addition of viscosity enhancers (e.g., hydrophilic cellulose derivatives, polyvinyl alcohol), permeability enhancers (cyclodextrins, chelating agents), or the reduction in the volume of the eye drop [3,4]. Тhese strategies help improve the ocular bioavailability only to a certain extent and, simultaneously, may induce adverse effects such as epithelial disruption (by permeability enhancers), blurred vision (by the viscosity enhancers approach), or lead to inaccurate dosing [5,6].

In this regard, the development of nanoscale drug delivery systems or platforms, such as in situ gels or contact lenses, are currently among the most exploited approaches to improve drug delivery in ocular therapeutics. Our study is based on both strategies, combining their individual advantages. Niosomes as nanocarriers provide the possibility to protect the encapsulated cargo from the enzymes present on the corneal surface, enhance drug solubility and stability, and release drugs in a controlled manner [7], whereas the in situ gelling system contributes toward a longer contact time and accurate dosing [8]. Additionally, niosomes’ distinctive vesicular structure—an inner aqueous compartment and an external bilayer membrane composed of non-ionic surfactants and cholesterol—enables the accommodation of drugs with different physicochemical characteristics [9].

The model drugs in the current study were plant-derived biologically active compounds, the lipophilic phytocannabinoid CBD (Mw 314.47 g/mol) and the hydrophilic catechin EGCG (Mw 458.372 g/mol). Their opposed nature, namely hydrophobic CBD (LogP = 6.3) and hydrophilic EGCG (LogP = 0.7), suggests different localizations in the niosomes in the membrane and the hydrophilic core, respectively. This, in turn, is a prerequisite for the high loading efficiency of both substances without potential competition during their encapsulation. The rational approach in the simultaneous loading of both substances into niosomes, on the one hand, solves the problems associated with the low aqueous solubility of CBD (0.012 g/L) and, on the other, optimizes the passage of highly hydrophilic EGCG (water solubility 5 g/L) through the diffusion cell barriers. In ophthalmology, CBD has been used in the therapy of glaucoma, keratitis, uveitis, and dry eye disease [10], whereas EGCG’s beneficial effects are mainly related to corneal oxidative damage [11] and dry eye disease [12]. Both phytochemicals differ in their mechanism of action in regard to *keratoconjunctivitis sicca*. CBD’s effects are mainly ascribed to its interaction with the receptor of the cannabinoid system (CB1), closely located near the transient receptor potential vanilloid-1 (TRPV1) receptor. The latter is activated following the release of nerve growth factor (NGF) in cases of corneal damage and ocular inflammation [10]. The therapeutic potential of EGCG is attributed to its antioxidant activity and anti-inflammatory effect, while the latter manifested as a decrease in the expression of pro-inflammatory cytokines, such as interleukin 1 (IL-1) and 6 (IL6) [12,13]. In the current study, the effect of dual loading of both compounds is estimated as potential synergetic antioxidant activity in the in vitro model of H_2_O_2_-induced oxidative damage, as well as the potential improved antibiofilm activity. To ensure longer corneal time, the dual-loaded niosomes were further included in the in situ gelling system.

In situ gelling systems are viscous liquid solutions capable of transforming into three-dimensional gel structures as a result of environmental stimuli (e.g., differences in temperature, pH value, or ionic composition) [8]. The current study exploits the last trigger mechanism. To the best of our knowledge, niosomes have not been incorporated into ion-sensitive in situ gels for ophthalmic application. Deacetylated gellan gum was selected as the gel-forming polymer due to its advantageous properties, such as biocompatibility and biodegradability, as well as superior gel strength and thermal stability (compared to the native polymer) [14,15].

The current study aims to develop and explore the potential of dual-loaded niosomal ion-sensitive in situ gel for the ocular delivery of CBD and EGCG. According to the authors’ knowledge, this is the first report on the simultaneous loading of both phytochemicals in niosomes, which will contribute to novel findings within this area. Additionally, their subsequent inclusion into in situ gels would further bring scientific value, evaluating the effect of vesicular dispersion on the gelling and rheological characteristics of in situ gels. A comprehensive physicochemical characterization of the nanocarriers and rheological evaluation of the in situ gelling system was performed, outlining the factors affecting their properties. The toxicity of the developed niosomal hybrid system was evaluated on HaCaT cells, and the antioxidant and antibiofilm activities of the dual-loaded phytochemicals were assessed.

## 2. Results and Discussion

### 2.1. Formulation and Characterization of CBD-Loaded and CBD-EGCG-Loaded Niosomes

The preliminary stage of our study included an optimization of the technological parameters of the method of preparation of niosomes (TFH method) by varying the rate of homogenization and duration of pulsatile sonication cycles. The homogenization rate throughout the experiment was selected as 150 rpm because the lower tested velocities (50 rpm and 100 rpm) led to the formation of aggregates on the bottom of the flask, which would negatively affect the subsequent hydration process and formation of physically stable and uniform dispersion. In terms of sonication time, it was estimated that the continuous sonication of niosomes for 2 min led to the formation of vesicles with sizes below 40 nm, which would adversely affect the entrapment efficiency of CBD and the retention of the nanocarriers on the ocular surface. The pulsatile sonication in a 20s on/ 10 s off mode for a period of 2 min resulted in the formation of homogeneous populations of niosomes with dimensions suitable for ocular delivery. The composition and physicochemical properties of the dual-loaded CBD-EGCG niosomes are presented in Table 1.

As evident from the obtained results, the type and molar ratio of surfactants affect the entrapment efficiency values of both phytochemicals. The highest entrapment for both drugs was estimated in formulation N3 based on equimolar ratios of Span 60 and Tween 60. This is probably due to the hydrophilic–lipophilic balance (HLB) of 9.8 of the resultant mixture, which facilitates the accommodation of both hydrophilic (EGCG) and lipophilic (CBD) compounds. Furthermore, the polar region in the molecule of Tween 60 enables the formation of hydrogen bonds with CBD, leading to its solubilization and, ultimately, the higher entrapment of the biologically active compound. The role of the surfactant concentration on the entrapment efficiency of CBD was also estimated in the study of Moqejwa et al., 2022 [16], which discusses the elaboration of CBD-loaded transfersomes. The authors reported that increasing the amount of polysorbate 80 leads to higher CBD entrapment efficiency, which may be attributed to the increased size of the vesicles and the larger area of the lipid bilayer available for the encapsulation of hydrophobic molecules [16].

The influence of the cholesterol content on the encapsulation efficacy was also investigated, and the results indicate that the increase in its concentration to 40% mol (formulations N1 and N2) does not affect the encapsulation of CBD, as nearly the same CBD EE% values as the formulations containing 30 mol% cholesterol were observed. Contrarily, the increase in cholesterol content to 40 mol% reduced the entrapped EGCG by nearly two times. The latter may be attributed to the reaching of the saturation capacity of the bilayer, above which a higher cholesterol content creates transient pores and leakage of the hydrophilic compound.

Concerning the influence of CBD as a component of membranes (N4), the presented results clearly demonstrate that its inclusion leads to membrane compaction and a decrease in vesicles’ size compared to empty vesicles (composition N6). EGCG entrapment efficiency was not significantly affected by cannabidiol concentration, a finding further supporting the different localizations in the vesicle structure. EGCG was successfully loaded into the Span 60-based niosomes intended for oral administration, with an entrapment efficiency of 40% [17]. A similar tendency was also estimated in the study by Yücel et al., in which EGCG-loaded ethosomes were prepared. The authors reported that increasing the lipid ratio (from 2% *w*/*v* to 4% *w*/*v*) reduces the entrapped EGCG (from 51.7% to 50.1%) [18]. In another study, Avadhani et al. prepared EGCG and hyaluronic acid dual-loaded transfersomes and estimated that increasing the amount of EGCG corresponds to higher entrapment efficiency values, but only to a certain extent (up to 10% *w*/*w*); afterward, a decrease in the EGCG entrapment was estimated, probably due to leakage of the phytochemical [19].

**Figure 1:**
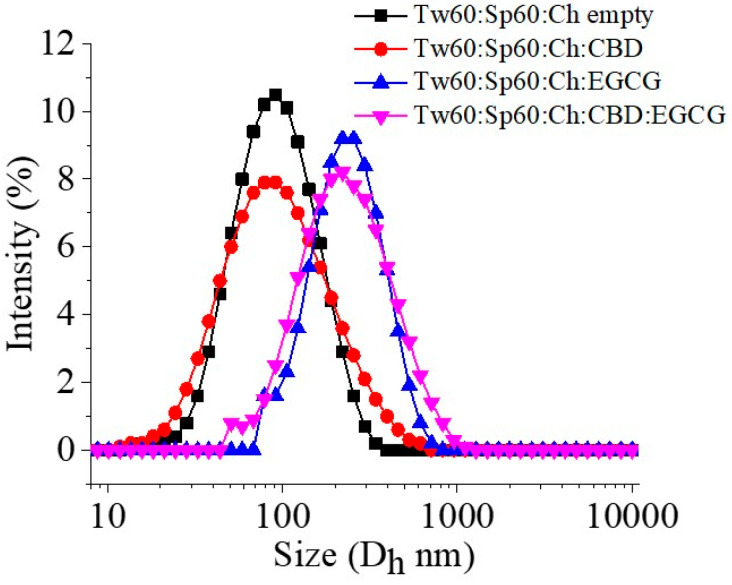
Size distributions of empty and drug-loaded niosomes.

Niosomal size was also affected by the inclusion of EGCG. The significant increase (*p* < 0.05) in mean diameter from 133 nm to 278 nm (compounds N6 and N5, respectively) is an indication of the successful loading of hydrophilic epigallocatechin (Figure 1). Zeta potential values of the developed niosomes ranged from −11 to −13 mV. The obtained negative values may arise as a result of the ionization of the free groups localized on the surface of the nanocarriers, as well as a result of the orientation of the hydroxyl groups with respect to water and the successive changes in the ionic charges [20].

The performed physicochemical studies revealed that the selected method of preparation and composition based on Span 60, Tween 60, and cholesterol (3.5:3.5:3 molar ratio (formulations N3 to N5) obtained reproducible populations of niosomes, characterized by high entrapment efficiencies for both phytochemicals and suitable sizes for better retention on the ocular surface.

### 2.2. Cryo-TEM Images of Niosomes

**Figure 2:**
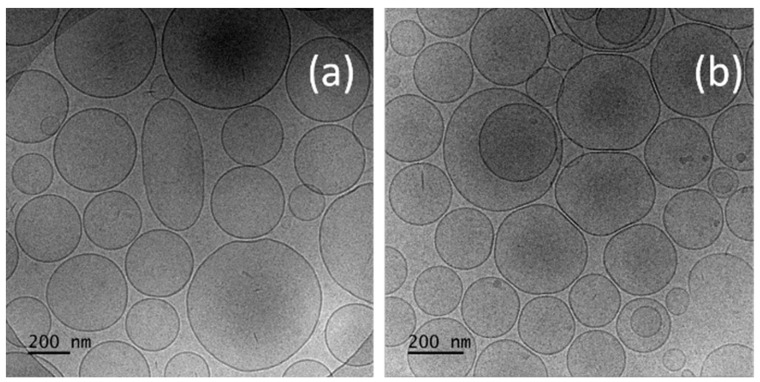
Cryo-TEM images of (**a**) empty niosomes (N6); (**b**) CBD-loaded niosomes (N5).

The morphology of developed vesicular carriers is assessed via cryo-TEM, and the relevant micrographs are shown in Figure 2.

As shown in the representative micrographs, the selected method of preparation (TFH method followed by pulsatile sonification) determines the formation of sphere-shaped vesicles with a distinctive bilayer membrane (t ≈ 4 nm). The blank niosomes exhibit an average size of 133 nm. The inclusion of CBD leads to a non-significant decrease in size (vs. the unloaded vesicles), with an average diameter of 124 nm (Figure 2b). The sizes based on cryo-TEM images are in accordance with the results obtained by dynamic light scattering analysis. After summarizing the results from the detailed physicochemical characterization of the prepared vesicles, formulation N3 (based on Tw60:Sp60:Chol (3.5:3.5:3 molar ratio)) was chosen as the optimal one and subjected to further evaluation.

### 2.3. Physical Stability Studies

An important aspect of the technological characterization of the elaborated nanocarriers is the monitoring of their storage stability. As shown in Table 2, no statistically significant changes in the size distribution pattern of niosomes were estimated at the end of the test period (30 days). A significant (*p* < 0.05) decrease in the entrapment efficiency values was estimated only for CBD at the end of the test period.

Based on the results from the conducted stability studies, it can be concluded that niosomes composed of Span 60/Tween 60/cholesterol (3.5:3.5:3 mol/mol) (N3) possess sufficient storage stability, which motivates their subsequent inclusion in the structure of an in situ gelling system.

### 2.4. Preparation and Characterization of In Situ Gelling Systems

A series of plain in situ gels were prepared to outline a composition with suitable gelling properties for the subsequent inclusion of niosomal dispersion. For potential ophthalmic application, it is important that the instilled system exhibits short gelation time and appropriate viscosity, ensuring its long residence time at the ocular surface while simultaneously allowing its uniform distribution without discomfort after administration. Plain in situ gels were prepared using gellan gum (G1–G6) and a combination of GG and hydroxypropyl methylcellulose (HPMC) (G7–G10) as a co-gelling agent. Gellan gum was selected as the main gelling agent due to its capability to undergo a phase transition from solution to gel state under the influence of the ionic composition of the medium (Ca^2+^, Na^+^, Mg^2+^ cations) and to form transparent, stable, and biodegradable gels. A possible mechanism of gelation may be the complexation reactions with cations and the occurrence of hydrogen bonds with water molecules, leading to the formation of double helical zones and, subsequently, a three-dimensional gel network [21]. HPMC, as an additional gelling agent, was chosen based on its excellent physicochemical properties and biocompatibility, with the assumption that it would allow the use of lower concentrations of gelling agents while preserving gelation time and capacity. The composition of different in situ gels is presented in Table 3.

All prepared plain in situ gelling systems represented clear, colorless viscous liquids at 20 °C, whereas the niosome-loaded gel formulations (G6N, G6N:CBD:EGCG) exhibited an opalescent to milky translucent appearance. After mixing the formulations with simulated tear fluid in a ratio of 40 μL:7 μL, their viscosity increased, leading to the formation of a soft gel at a concentration of gellan gum above 0.3% and of gellan gum and HPMC above 0.2% and 0.25%, respectively. However, the results for compositions based on the combination of both polymers showed that the inclusion of HPMC did not increase the gelling capacity of the developed systems, and practically no hard gels were observed. Among the tested plain models, optimal gelation characteristics (lowest time for gelation and sufficient gelling capacity) were for model G6 (based on 0.6% gellan gum); therefore, this was selected as optimal for successive inclusion of niosomal dispersions either empty (blank gel—G6N) or dual loaded (G6N:CBD:EGCG gel), allowing us to outline the effect of the formulation variables on the gelling properties of the system. As evident from the presented data, the gelling capacity was not affected by the inclusion of niosomes, which indicates that the type and concentration of the polymer used as a gelling agent are the main factors influencing the sol-to-gel transition of the systems. However, a slight reduction in the gelation time was observed upon the addition of dual-loaded vesicles, which may be related to the formation of more hydrogen bonds within the system as a result of the presence of both phytochemicals.

### 2.5. Rheological Properties of In Situ Gels

**Figure 3:**
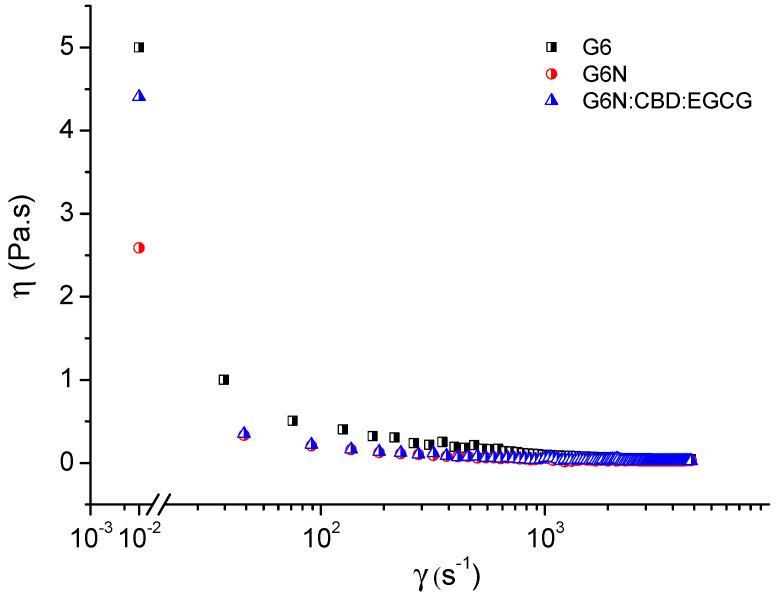
Viscosity as a function of the shear rate of G6, G6N, and G6N:CBD:EGCG formulations at 25 °C.

Firstly, the viscosity of drug-loaded formulations (G6N:CBD:EGCG) before the addition of simulated tear fluid (STF) was assessed. As mentioned above, the liquid state of an in situ gelling delivery system prior to administration is necessary because it allows free flow and the ability to form droplets. The measurements were carried out at 25 °C in the CR (Controlled Rate) mode, changing the shear rate from 0.01 to 4500 s^−1^. The viscosity behavior of G6N:CBD:EGCG was compared with that of the pure gel base (formulation G6) and blank niosomal gel containing empty vesicles (formulation G6N). After mixing with STF (40 µL:7 µL *v*:*v* ratio), all three formulations underwent transition from sol to gel due to the chemical bonding of the carboxylate groups with the calcium ions present in the STF [22]. The three samples exhibited a shear-thinning behavior, and a significant drop in viscosity was observed at a relatively low shear rate (Figure 3). This observed pseudoplastic flow of the prepared formulations is preferable for ophthalmic application as it promotes considerably less resistance to blinking and, thus, a higher acceptance than the viscous Newtonian formulation [23]. Adding niosomal nanocarriers (empty and drug-loaded) to the polymer solution decreased the viscosity of the formulations; however, the viscosity values at a shear rate of 4500 s^−1^, which simulates the conditions under the shear of the eyelid [24], were identical. The measured shear viscosity of 25 mPa·s is very close to that of some commercial eye drops, which gives us a reason to conclude that the developed formulation is suitable for ocular administration [25].

**Figure 4:**
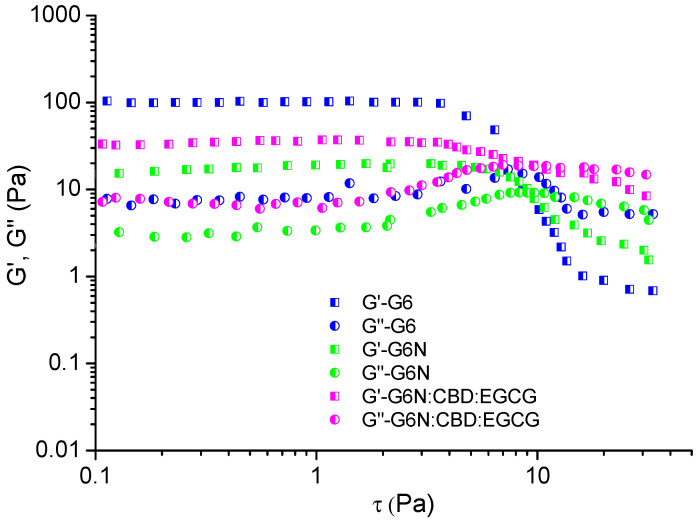
Variation in elastic (G′) and loss (G″) moduli as a function of shear stress (τ) of G6, G6N, and G6N:CBD:EGCG formulations. All measurements were carried out at 35 °C.

Next, oscillatory tests were conducted to determine whether the formulations were able to transition from a liquid to a gel state when STF was added to the composition [26]. The STF was blended with the studied samples on the rheometer plate at 35 °C, and then the measurements were taken at the same temperature. Figure 4 presents the oscillation stress sweep plots of samples G6, G6N, and G6N:CBD:EGCG. Usually, physical hydrogels are characterized by a linear viscoelastic region (LVR), where the elastic (G′) and loss (G″) moduli are independent of the stress value, and the elastic component dominates the viscous one (G′ > G″) [27]. In our case, the three samples exhibited gel-like behavior, with a well-defined plateau on the left side of the plots. Indeed, in the range with the lowest stress values, G′ was notably higher than G″, which is direct proof of the formation of a three-dimensional structure (network). As expected, at a higher shear, the viscous portion became dominant, and the material began to flow. It seems that niosomal aggregates caused a slight shift of the yield stress (the limit of LVE region) and flow point (G′ = G″) toward higher values and reduced the G′ of the system in the LVE region. Overall, the oscillatory tests confirmed that the G6N:CBD:EGCG formulation possesses suitable rheological characteristics to enhance the residence time on the ocular surface.

### 2.6. Drug Release Studies

**Figure 5:**
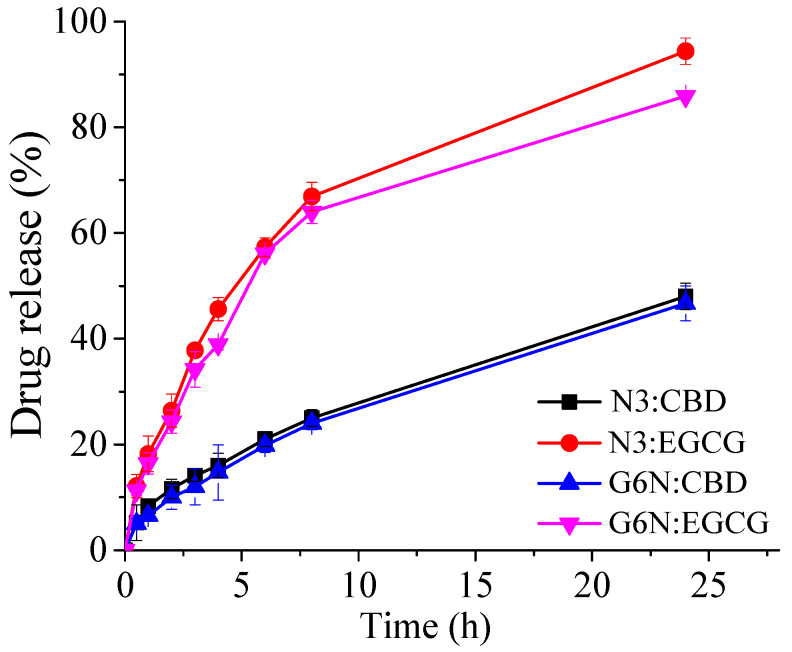
In vitro release profile of CBD and EGCG from optimal niosomal formulation (N3) and its hybrid in situ gelling system (G6N:CBD:EGCG). N3:CBD, G6N:CBD denote cannabidiol release from niosomal suspension and hybrid niosomal gel, respectively, whereas N3:EGCG and G6N:EGCG represent the release profiles of epigallocatechin-3-gallate from niosomes and its hybrid niosomal gel formulation. Each value is presented as mean ± SD (n = 3).

The release profiles of CBD and EGCG from niosomes and in situ gelling systems are illustrated in Figure 5.

As evident from the presented data, drug release from niosomes is a two-phase process, comprising an initial higher release rate (i.e., burst effect) and a subsequent sustained release. The observed in vitro profile is characteristic of these types of nanocarriers; however, it is noteworthy to mention that the process is greatly affected by the physicochemical properties of the loaded phytochemicals. In this regard, the hydrophilic EGCG is characterized by a higher release rate compared to the hydrophobic CBD. A slight reduction in drug release was observed upon the inclusion of niosomes into the in situ gelling system, which may be attributed to the barrier function of the three-dimensional polymer network, restricting the diffusion of the entrapped compounds. A similar tendency was estimated in other relevant studies [28,29], irrespective of the type of incorporated nanocarrriers, e.g., nanoparticles or vesicular systems. In their study, Abbas et al. prepared oxytetracycline-loaded nanoparticles included in thermosensitive (Poloxamer 407-based) in situ gel. The observed lower drug release from the in situ gelling systems may be explained by the crosslink formation in the Poloxamer matrix and the resulting obstruction thereof, which further emphasizes the role of the drug delivery platform in the release process [28].

To evaluate the release mechanism of CBD and EGCG from niosomes and in situ gels, the data from the release studies were fit to different kinetic models, and the obtained results are presented in Table 4. The calculated coefficients of determination indicated that EGCG release from niosomes and the in situ gel followed first-order kinetics, whereas CBD release may be best attributed to the Korsmeyer–Peppas model. The obtained diffusion exponent values above 0.5 and less than 1 indicate that CBD release follows a non-Fickian diffusion, which implies that the CBD release mechanism is rather complex where, in addition to the diffusion, the possible redistribution of the released drug in the polymer 3D network of the gel or onto the niosomal membranes due to its hydrophobic nature may contribute simultaneously.

### 2.7. Microbiological Studies

#### Minimal Inhibitory Concentrations and Antibiofilm Activity of Ocular Gel with Epigallocatechin and Cannabidiol

The MIC values of the tested ocular gel revealed a moderate in vitro effect against the Gram (+) strains from the *S. aureus* species. The visible bacterial growth of both strains was inhibited after exposure to a 1:8 dilution that contains EGCG:CBD in a ratio of 0.125:0.0625 mg/mL. The metabolic activity of the bacteria was not affected. This points to a bacteriostatic effect of the combination.

**Figure 6:**
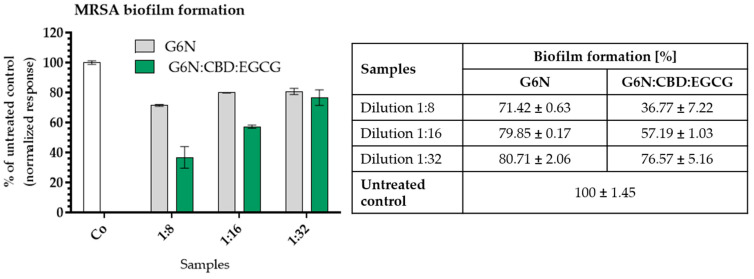
Quantitative evaluation of MRSA biofilm formation after exposure to blank (G6N) or hybrid niosomal drug-loaded gel G6N:CBD:EGCG (1/0.5 mg/mL). Legend: Co—untreated control; Dilution 1:8 = 0.125/0.06125 mg/mL; Dilution 1:16 = 0.0625/0.03125 mg/mL; Dilution 1:32 = 0.03125/0.0156 mg/mL. Each value is presented as mean ± SD (n = 4).

The quantitative evaluation of the biofilm inhibition reveals approximately 60% inhibition of the MRSA adhesion compared to the untreated control (Figure 6). The effect is concentration-dependent and decreases with increased dilution. The results show (Supplementary Materials, Tables S1 and S2) that the plain niosomal gel slightly inhibits the biofilm formation, but there is no significant difference between the three dilutions of the gel (G6N). On the contrary, there is a significant difference (*p* < 0.002) between the dilutions of drug-loaded G6N:CBD:EGCG formulation, the untreated control, and the dilutions, showing the concentration-dependent biofilm inhibitory effect of the combination. A dilution of G6N:CBD:EGCG inhibits the MRSA biofilm formation with approximately 60%, dilution 1:16 with around 42%, and dilution 1:32 with ca. 20% (which is the same as that of G6N).

CBD is known for its potential to prevent attachment of Staphylococci adhesion to keratinocytes [30]. The antibacterial effect of epigallocatechin gallate against *S. aureus* is related to the modulation of gene expression responsible for the membrane transport, which results in decreased membrane potential of the bacterial cell and impaired adhesion capacity [31]. Therefore, we investigated the antibiofilm potential of the combination loaded in an ocular gel starting with concentrations of ½ MIC (dilution 1:8) and lower (dilutions 1:16 and 1:32) against MRSA. As shown in Table 5, the G6N:CBD:EGCG gel significantly inhibits the adhesion of the pathogen compared to the blank niosomal gel (G6N). To our knowledge, this is the first investigation of the antibacterial effect of such a combination loaded in an ocular gel against MRSA. Our hypothesis is based on published data about the antibacterial potential and mode of action of CBD and EGCG [32,33,34]. CBD has a complex mechanism of action, expressed in the inhibition of peptidoglycan, DNA, and RNA synthesis [32]. EGCG damages cell membranes and increases their permeability. The results obtained in our study do not differ from the reports in the literature. The reported MICs of CBD for Gram-positive bacteria range from 1 to 5 µg/mL [35,36,37,38]. The incubation period for the MIC and biofilm assay is 24 h, and around 50% of the CBD is released from the ocular gel for that time period (See Figure 5). Therefore, the effective concentration of CBD in the combination in the biofilm assay used in our study is ca. 60 µg/mL. During the first 5 h after treatment, less than 20% of CBD is released. The slower release of CBD could be the reason for the higher concentration needed to achieve an inhibitory effect different from the reported data of other authors who worked with the pure substance. Nevertheless, further investigations are needed to prove this hypothesis, which could be an object of another study. The active concentrations of EGCG reported in the scientific literature are between 60 and 400 µg/mL [33,34,38]. The effective concentration of EGCG in our study with the combination of CBD is 125 µg/mL, which is in line with the published data. Considering the fact that ca. 40% of EGCGs are released within the first 5 h and up to 80% of EGCG after 24 h, we can assume that the effective biofilm inhibitory concentration of EGCG is ca. 50 µg/mL, which is lower than the reported MIC values in the studies cited above. Based on the presented result, we can assume that the tested combination is promising for the inhibition of MRSA biofilms and will be beneficial for the treatment of concomitant bacterial infections.

### 2.8. In Vitro Toxicity Evaluation

Ocular toxicity is an essential factor in the safety evaluation of different products, including drugs and drug delivery systems [39]. In particular, toxicity studies of nanocarriers are a crucial component of preclinical safety evaluations. Niosomes offer several advantages as a drug delivery system, among them a favorable safety profile [40]. They are considered to possess low toxicity due to their non-ionic nature. Non-ionic surfactants exhibit greater compatibility and lower toxicity compared to anionic, amphoteric, or cationic surfactants [41]. Nevertheless, the segregation of non-ionic surfactants may lead to toxicity. Furthermore, when niosomes are employed to target and reduce drug side effects, toxicity can also result from the location and concentration of the released drug [42].

**Figure 7:**
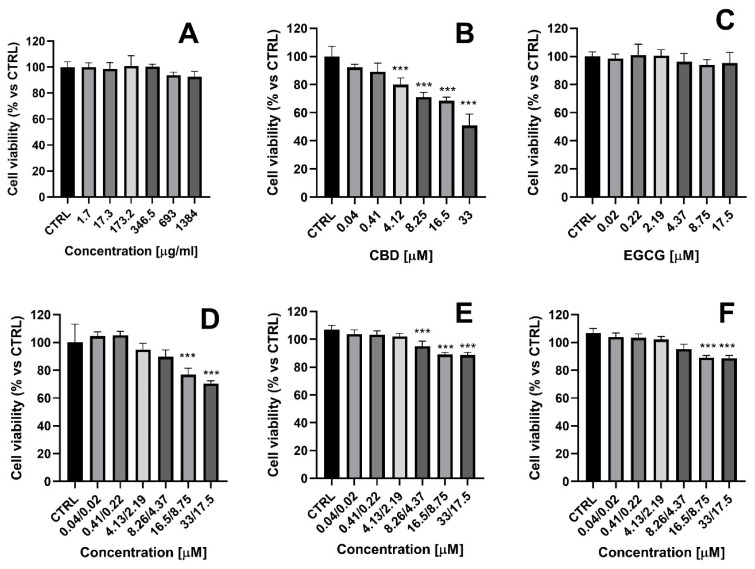
Cytotoxicity on HaCaT cells of: (**A**) empty niosomes (N6); (**B**) free cannabidiol (CBD); (**C**) free epigallocatechin (EGCG); (**D**) combination of free cannabidiol and free epigallocatechin (CBD + EGCG); (**E**) dual-loaded CBD and EGCG vesicles (N:CBD:GCG, formulation) niosomes and (**F**) niosomal in situ gel based on double-loaded niosomes (G6N:CBD:EGCG), measured by MTT assay. All groups were compared statistically vs. untreated controls by one-way ANOVA with Dunnet’s post hoc test. The results are expressed as means ± SD of triplicate assays (n = 8). *** *p* < 0.001 vs. control (CTRL, untreated control cells).

In the present study, the cytotoxicity effects of unloaded niosomes at various concentrations (1.7–1384 µg/mL) were evaluated on HaCaT cells, aimed at establishing the safety of empty nanocarriers (Figure 7A). HaCaT cells (human keratinocytes) were selected to evaluate the toxicity of the test solutions because it is a suitable corneal epithelial model commonly used for eye toxicity assessment [43]. We found that empty niosomes did not show cytotoxic effects in human keratinocytes after 24 h of treatment. Next, we evaluated the effects of free drugs and their niosomal formulations on HaCaT cell viability (Figure 7B–F). CBD was not cytotoxic in concentrations up to 4.12 µM, but at the highest concentration (33 µg/mL), it decreased cell viability by 50% (Figure 7B). These results are consistent with data reported by Krajka-Kuźniak et al., who demonstrated an IC_50_ of cannabidiol of 31 µg/mL in the HaCaT cell line (24 h) [44]. The effects of EGCG (0.02–17.5 µМ) on the HaCaT cell line were also investigated. Unlike CBD, free EGCG did not exhibit any cytotoxic effects. The combination of non-loaded CBD and EGCG showed slight cytotoxicity only at the highest concentration of the active substances, but the effect was less pronounced than that for free CBD tested at equimolar concentrations. Notably, the dual loading of CBD and EGCG in niosomes (N:CBD:EGCG) and the hybrid gelling system (G6N:CBD:EGCG) showed a good safety profile. We observed only a slight decrease (<20%) in HaCaT cell viability at the highest combination concentrations, which is considered slight cytotoxicity, according to ISO10993-5:2009 [45].

### 2.9. Protective Effects of Test Compounds in a Model of H_2_O_2_-Induced Oxidative Damage In Vitro

**Figure 8:**
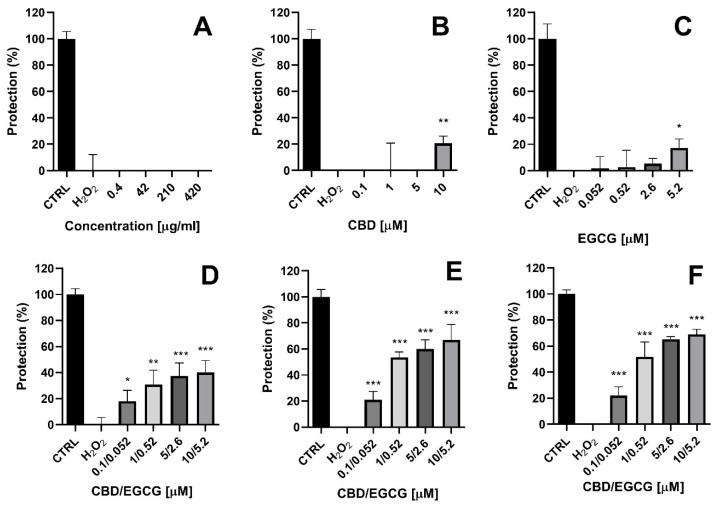
Protective effects of (**A**) empty niosomes; (**B**) free CBD; (**C**) free EGCG; (**D**) combination of free CBD and free EGCG (CBD + EGCG); (**E**) dual-loaded CBD and EGCG (N:CBD:EGCG) niosomes and (**F**) niosomal in situ gel based on double-loaded niosomes (G6N:CBD:EGCG) in a H_2_O_2_-induced damage model in human keratinocyte HaCaT cell line. The results are expressed as means ± SD of triplicate assays (n = 8). ANOVA with Dunnett’s post-test. * *p* < 0.05; ** *p* < 0.01; *** *p* < 0.001 vs. H_2_O_2_ CTRL (untreated control cells); H_2_O_2_ cells treated with H_2_O_2_ (200 µM).

CBD and EGCG have been shown to possess protective anti-inflammatory and antioxidant effects in different experimental in vitro models. For example, CBD (10 μM) was shown to protect HaCaT cells by alleviating H_2_O_2_ (200 μM)-induced cytotoxicity and by reducing reactive oxygen species formation [46]. Furthermore, Ugartondo et al. demonstrate the protective effects of epicatechin and its derivatives on red blood cells and the HaCaT cell line in response to exogenous H_2_O_2_ exposure [47]. Therefore, we next evaluated the effects of dual-loaded niosomes (N:CBD:EGCG) as well as the hybrid niosomal gelling system (G6N:CBD:EGCG) in a model of H_2_O_2_-induced oxidative stress in HaCaT human keratinocytes. H_2_O_2_ generates oxygen radicals that directly cause oxidative damage to normal human keratinocytes. Increasing evidence suggests that oxidative stress-induced cytotoxicity in keratinocytes plays a role in the pathogenesis of skin and ocular diseases [48]. The experimental design involved pre-treating the cells with test samples for 24 h. Then, oxidative stress was induced by 200 μM H_2_O_2_ for 24 h, and cell viability was assessed using the MTT assay. For comparison, the effects of the empty niosomes (N6) applied at concentrations ranging from 0.4 to 420 μg/mL were also evaluated. The exposure of HaCaT cells to 200 μM H_2_O_2_ (24 h) resulted in a loss of approximately 55% of cell viability. Unloaded niosomes (N6) did not exhibit protective effects in the oxidative damage model (Figure 8A). Incubation of the cells with unloaded CBD (10 μM) and EGCG (5.2 μM) caused statistically significant protection, expressed as preservation of cell viability by 20% and 17%, respectively (* *p* < 0.05, ** *p* < 0.01). An increased protective effect was observed in the combination of the free substances (CBD + EGCG), where statistically significant preservation of cell viability was seen across the entire concentration range (0.1/0.052 μM–10/5.2 μM). However, it should be noted that the highest protection was observed in the dual-loaded niosomes (N:CBD:EGCG), as well as the hybrid gelling system G6N:CBD:EGCG. At concentrations of 0.1/0.052 μM, 1/0.52 μM, 5/2.6 μM, and 10/5.2 μM, the protection shown by dual-loaded niosomes was, respectively, 21%, 54%, 60%, and 67%, and 22%, 51%, 65%, and 69% for the hybrid gelling system compared to cells treated with H_2_O_2_ (0% protection) (** *p* < 0.01, *** *p* < 0.001). Therefore, we found that dual loading of CBD and EGCG enhances their protective properties in the oxidative stress model, and the inclusion of niosomes in the hybrid gelling system preserves these effects.

## 3. Conclusions

CBD and EGCG-loaded niosomes were elaborated and further formulated into a hybrid in situ gel based on ion-sensitive gellan gum, which was evaluated as a potential drug delivery platform for eye administration. A hybrid in situ gel formulation composed of 0.6% *w*/*w* gellan gum and dual-loaded CBD:EGCG niosomes based on Tw60:Sp60:Chol (3.5:3.5:3 molar ratio) was selected as most suitable for ophthalmic administration due to its high gelling capacity, short time for gelation (35 s), typical shear-thinning behavior at physiological temperature, and ability to release loaded drugs in a controlled manner. In addition, the proven inhibitory effect on bacterial biofilm formation and the pronounced protective antioxidant potential gave us reason to conclude that the developed hybrid niosomal in situ gel is a promising platform for delivering CBD and EGCG to the eye.

## 4. Materials and Methods

### 4.1. Materials

CBD was generously provided by PBG GLOBAL, Sofia, Bulgaria. Non-ionic surface-active agents (Span 20, Span 60, Span 80, Tween 60), cholesterol, epigallocatechin-3-gallate, gellan gum (Gelrite^®^), and hydroxypropyl methylcellulose (120–220 cps) were obtained from Sigma-Aldrich (FOT, Sofia, Bulgaria). HaCaT cell line (human keratinocytes) was acquired from the European Collection of Cell Cultures (ECACC, Salisbury, UK). The cells were cultured in DMEM (Dulbeco’s modified essential medium) (Manassas, VA, USA), supplemented with 10% heat-inactivated FBS (Sigma-Aldrich Co. LLC, St. Louis, MO, USA), 1 mM L-glutamine (Sigma-Aldrich Co. LLC, St. Louis, MO, USA. The cells were passaged in 75 cm^2^ flasks each 3–5 days via trypsinization (StableCell Trypsin solution, Sigma-Aldrich Co. LLC, St. Louis, MO, USA). MTT salt and H_2_O_2_ were bought from Sigma-Aldrich Co. LLC, St. Louis, MO, USA). The chemicals used for the preparation of simulated tear fluid (STF) were of analytical grade: sodium chloride (6.8 g), sodium hydrogen carbonate (2.2 g), calcium chloride dehydrate (0.084 g), and potassium chloride (1.4 g) in 1 L purified water.

### 4.2. Methods

#### 4.2.1. UHPLC-DAD Analysis

The UHPLC-DAD evaluations were performed using a Thermo Scientific Dionex UltiMate 3000 (Thermo Fisher Scientific, Waltham, MA, USA) analytical system equipped with a Dionex UltiMate 3000 RS Pump (LPG-3400RS), Dionex UltiMate 3000 RS Autosampler (WPS-3000TRS), Dionex UltiMate 3000 RS Column Compartment (TCC3000RS) and Dionex UltiMate 3000 Diode Array Detector (DAD-3000). The separation was performed on an Acquity UPLC BEH C18 column (2.1 × 100 mm, 1.7 μm) (Waters), with a mobile phase composed of A 0.1% formic acid and B acetonitrile. The applied gradient was: 0 min 5% B, 5 min 20% B, 10 min 95% B, 15 min 95% B. Thereafter, the system was set to the initial condition and equilibrated for 5 min. The utilized flow rate was 300 μL/min with an injection volume of 1 μL. The UV wavelength was 280 nm. The analytical process was controlled by Chromeleon software, version 7.2.

##### Quantitative Analysis

The quantification of CBD and EGCG was conducted using the external standard method. Standard calibrations of both CBD and EGCG were made at seven data points in the concentration range of the respective analytes in accordance with the level anticipated in the samples. The solutions containing 0.85, 0.43, 0.21, 0.11, 0.05, 0.03, and 0.003 mg/mL of CBD and 1.95, 0.98, 0.49, 0.24, 0.12, 0.06, and 0.006 of EGCG were prepared in methanol. Each concentration was measured in triplicate, and the corresponding peak area was detected at 280 nm. The main parameters of the elaborated analytical method, such as limit of detection, limit of quantification, and regression equation, are given in Table 6.

#### 4.2.2. Preparation of CBD-EGCG-Loaded Niosomes

Niosomes (Table 7) were prepared using the thin film hydration method with successive sonication. Briefly, accurately weighted amounts of non-ionic surfactant and cholesterol (30 µmol/mL total) were dissolved in chloroform in a round-bottomed flask. Afterward, a methanolic solution of CBD (3 µmol/mL) was added, and the resultant mixture was rotary evaporated (150 rpm) until the formation of a uniformly distributed thin lipid film. The hydration step was performed at 60 °C for 1 h using deionized water for the blank and CBD-loaded niosomes or solution of EGCG in deionized water (2 mg/mL corresponding to 4 μmol/mL) for the dual-loaded vesicles. The obtained dispersions were subjected to probe (2 mm diameter) sonication (Bandelin Sonoplus HD2200, Berlin, Germany) at 30% amplitude for 2 min (continuous or pulsatile 20 s on/10 s off). Afterward, to separate the non-encapsulated drugs, the niosomal suspensions were subjected to gel filtration through Sephadex G50 columns equilibrated in deionized water. The filtered niosomes were collected and kept at 4–6 °C until further analysis

#### 4.2.3. Cryogenic Transmission Electron Microscopy (cryo-TEM)

The structure of developed niosomes was evaluated using cryogenic transmission electron microscopy (cryo-TEM). Then, 3 μL of tested niosomal formulation was placed on the grid and spread in a thin film by blotting procedure and immediate freezing in liquid ethane using a fully automated blotting device Vitrobot Mark IV (Thermo Fisher Scientific, Waltham, MA, USA). Thereafter, the samples were transferred to a Tecnai F20 X TWIN microscope (FEI Company, Hillsboro, OR, USA) and examined at −178 °C and 200 kV acceleration voltage. The images were recorded using a Gatan Rio 16 CMOS 4k camera (Gatan Inc., Pleasanton, CA, USA) and analyzed via the relevant software (Gatan Inc., Pleasanton, CA, USA).

#### 4.2.4. Size and Zeta Potential Measurements

Dynamic light scattering analysis (DLS) was used to assess the size, size distribution, and polydispersity index (PDI) of the developed nanocarriers via ZetaSizer NanoZS (Malvern Instruments, Malvern, Worcestershire, UK). The measurements were taken three times at 25 °C with a scattering angle of 175°, and the measured hydrodynamic diameter indicated the mean vesicles’ size.

The zeta potential of vesicular dispersions was evaluated using ZetaSizer NanoZS (Malvern Instruments, Malvern, Worcestershire, UK) based on the electrophoretic light scattering. The measurements were performed in triplicate in deionized water at 25 °C, scattering angle of 175°, laser operating at 633 nm.

#### 4.2.5. Entrapment Efficiency

The prepared drug-loaded niosomes were subjected to gel filtration through a Sephadex G50 column (Pharmacia, Uppsala, Sweden). Afterward, the entrapment efficiency of both compounds was determined according to the following protocol. A precise volume of 100 μL of niosomal sample was dissolved in 1000 μL isopropanol, vortexed for 30 s, and was subjected to UHPLC-DAD. Peak identification was based on the comparative evaluation of the times for retention and UV spectra of the sample and reference standards. Triplicate analyses were performed for each sample, and the peak area was detected at 280 nm.

#### 4.2.6. Stability Studies

The stability of niosomes was determined by storing the samples for one month in a refrigerator (4 ± 2 °C). Afterward, the formulations were tested for potential alterations in their size, polydispersity index, and drug entrapment efficiency.

#### 4.2.7. Statistical Analysis

The obtained data are expressed as mean values ± standard deviation. Analysis of variance (ANOVA): Single Factor was performed in Excel to assess the results of niosomal size, PDI, and entrapment efficiency with the probability value (*p* < 0.05) considered to be statistically significant.

### 4.3. Preparation and Characterization of Plain and Niosomal Ion-Sensitive In Situ Gels

Required quantities of gellan gum (0.1, 0.2, 0.3, 0.4, 0.5, 0.6, *w*/*v*) were dispersed in deionized water and heated at 90 °C under constant stirring via a magnetic stirrer (350 rpm) for 15 min. Afterward, the dispersions were left to cool down at ambient temperature. In the formulations containing HPMC (0.25 and 0.5% *w*/*v*), the polymer was added gradually to the gellan gum aqueous dispersions during the stirring process.

Plain in situ gel formulations were evaluated for their gelling capacity by mimicking the conditions upon ocular instillation. For this purpose, the formulations were mixed with STF in 40:7 ratios (40 µL represents the applied volume of the formulation, whereas 7 µL equals the volume of tear fluid) on a watch glass. The gelling capacity was evaluated at ambient temperature for the ocular surface (35 °C). Simulated tear fluid was freshly prepared for the experiments by dissolving 6.8 g sodium chloride, 2.2 g sodium hydrogen carbonate, 0.084 g calcium chloride dehydrate, and 1.4 g potassium chloride in 1 L purified water [49]. The gelling capacities of the formulations were visually assessed in accordance with the following criteria: (-) no gelation, (+) slow weak gelation, (++) instant gel formation, lasting for 2–3 h, (+++) instant gelation lasting for an extended time (6–8 h) [1]. The time when no fluidity of the samples was observed upon inversion was denoted as gelation time.

Plain in situ gel formulation characterized by sufficient gelling capacities was used for the inclusion of CBD and EGCG-co-loaded niosomes. Niosomal ion-sensitive gels were prepared using deionized water and niosomal dispersion (1:1 *v*/*v*) as the vehicle. Initially, the polymer(s) were dispersed in half of the volume of deionized water required for gel formation as described above; afterward, niosomal dispersion was added and gently stirred until the formation of a homogenous solution, maintaining the polymers’ concentration in all samples.

### 4.4. Evaluation of Rheological Properties of In Situ Gelling Systems

Dynamic rheological measurements were conducted with a RheoStress 600 rheometer (Thermo Scientific HAAKE, Waltham, MA, USA). The tests were conducted with a parallel plate geometry (top plate diameter = 20 mm; gap = 1 mm). The viscosity curve was obtained in CR mode (Controlled Rate) at 25 °C. Storage and loss moduli were determined in CS (controlled stress) mode at 37 °C and a constant frequency of 1.000 Hz.

### 4.5. In Vitro Release Studies

The release profiles of CBD and EGCG from the tested formulations were investigated using the dialysis method. In brief, 4 mL of the niosomes or in situ gel prepared thereof (corresponding to 3.4 mg CBD and 4 mg of EGCG, based on the obtained entrapment efficacy) were placed in a dialysis bag (SpectraPor, 10,000 Da cut off, Spectrum Laboratories, Inc., Rancho Domingez, CA, USA), and set in a vessel containing a 50 mL volume of STF (pH 7.4) as acceptor medium at 35 °C. Samples of the medium were taken at specific time intervals and analyzed using a validated HPLC method.

For a more precise interpretation of the dissolution test data, a non-linear regression analysis was applied where the collected release data were tailored to various kinetic equations, namely: zero-, first, Higuchi, and the Krosmeyer–Peppas models using DDSolver—a freely available Excel plug-in software version 1.0 [50].

### 4.6. Microbiological Studies

#### 4.6.1. Bacterial Strains and Culture Conditions

To the best of our knowledge, in the current study, the antibacterial activity of the ocular gel containing niosomes loaded with EGCG and CBD in a ratio of 2:1 on the two bacterial strains: *Staphylococcus aureus* (ATCC 29213, American Collection of Microorganisms and Cell Cultures, Manassas, VA, USA), and *Escherichia coli* (ATCC 35218) was investigated for the first time. The selected microorganisms are the most frequently tested in antimicrobial activity evaluations in accordance with the standard ISO 20776-1 Part 1 [51]: Reference method for testing the in vitro activity of antimicrobial agents against rapidly growing aerobic bacteria involved in infectious diseases. The antibiofilm effect of the gel was evaluated on the strain *Staphylococcus aureus*—MRSA (NBIMCC 8327—resistant to methicillin and oxacillin, National Bulgarian Collection for Microorganisms and Cell Cultures, Sofia, Bulgaria). Strains were cultured on Mueller–Hinton broth/agar (MHB/MHA—#M391-500G/#M173-500G, Himedia, Mumbai, India) at 37 °C.

#### 4.6.2. Broth Microdilution Test

The minimum inhibitory concentrations (MIC) of the ocular gel were evaluated by the broth microdilution method according to ISO 20776-1:2019 [51]. Shortly, a bacterial dispersion with a density of 10^8^ (colony forming units) CFU/mL (OD600) was set in MHB from 18 h bacterial culture of the relative microorganism and then brought to a concentration of 5 × 10^5^ CFU/mL. Two time serial dilutions were prepared in triplicate in 96-well plates to a volume of 50 µL. MHB was utilized as a diluent and negative control. An aliquot of the bacterial dispersion was supplemented to each well, and the plates were incubated for 24 h at 37 °C. The lowest concentration that prevented noticeable bacterial progression is defined as the MIC. Penicillin G #B0500000, Merck KGaA, Darmstadt, Germany) in concentration 0.004–2 mg/L and gentamicin #11520506, Gibco, Therma Fisher Scientific, Wathman, MA, USA) in concentrations of 0.008–4 mg/L were used as reference antibiotics (positive controls). EUCAST (European Committee for Antimicrobial Susceptibility Testing) requirements were followed for reporting and discussion of the results.

#### 4.6.3. Evaluation of Bacterial Metabolic Activity

The metabolic activity of the bacterial strains treated with the ocular gel was measured at the 24th hour with MTT dye (#M2128-1G, Merk-Sigma Aldrich, Steinheim, Germany), which is reduced by the membrane-located bacterial enzyme NADH: ubiquinone reductase (H + -trans-location) to insoluble violet formazan crystals. For this purpose, the protocol of Wang et al. was applied [52] with some alterations. Shortly, 10 µL MTT 5 mg/mL in PBS (Dubecco’s phosphate-buffered saline, #D8537, Merck-Sigma Aldrich, Steinheim, Germany) was added to each sample. The samples were homogenized well and incubated for 60 min at 37 °C. The resulting formazan crystals were liquefied with an equal volume of 2-propranol containing 5% formic acid (HCOOC, Chimspektar OOD, Sofia, Bulgaria). The absorbance was measured at λ = 550 nm (Eliza reader Lx800, Bio-Tek Instruments Inc., Winooski, BT, USA) against a blank solution containing the respective volumes of MHB, MTT, and solvent. As a referent filter for the absorbance, one with a wavelength λ = 690 nm was used.

#### 4.6.4. Determination of Anti-Biofilm Activity on MRSA

The ocular gel was tested for the potential to prevent MRSA biofilm formation following the procedure of Stepanovic et al. [53]. Two time serial dilutions (100 µL/well) of the gel starting from dilution 1:4 (EGCG:CBD—0.25:0.125 mg/mL) were set in 96-well polystyrene tissue culture plates in BHI broth with 2% glucose (*w*/*v*). Bacterial inoculum was prepared in the same manner as described above for BMD, and a 100 µL aliquot was added to each well. Cells were incubated at 37 °C for 24 h under static conditions. Afterward, the planktonic cells were separated by three-fold washing with PBS (200 µL/well). The residual cells were fixed with 200 µL methanol (Merck-Sigma-Aldrich, Steinheim, Germany #34860-1L-R) for 15 min, air dried, and stained with 0.1% aqueous solution of crystal violet (#611 35, Merck-Sigma-Aldrich, Steinheim, Germany) in 100 µL/well. The plate was rinsed with tap water and air dried. Biofilm formation was visualized under microscope observation (40×). The biofilm-bound dye was solubilized with 160 µL 33% acetic acid (#8187551000, Merck-Sigma-Aldrich, Steinheim, Germany), and the OD of each well was measured at 550 nm (referent filter 690 nm). The biofilm inhibition was presented as a percentage of the untreated control with the GraphPad Prism software after data normalization. The statistical evaluation of the MRSA biofilm absorbance was performed using two-way ANOVA (GraphPad Prism version 9.0.0 for Windows, GraphPad Software, Boston, MA, USA). A *p*-value < 0.05 was considered for a statistically significant difference.

### 4.7. Culture Cell Line HaCaT and Cell Viability Assay

Human keratinocyte HaCaT cells were acquired from the European Collection of Cell Cultures (ECACC, Salisbury, UK) and cultured under specific conditions. The cells were grown in Dulbecco’s Modified Eagle Medium (DMEM) supplemented with 4.5 g/L glucose, 10% fetal bovine serum, and 2 mM L-glutamine. They were grown at 37 °C in an atmosphere of 5% CO_2_. Routine culturing was conducted in 75 cm^2^ culture flasks, providing an optimal environment for growth and maintenance.

Once the cells reached approximately 80% confluence, they underwent several sequential treatments. First, the cells were harvested using a trypsin/EDTA solution. Then, they were seeded into 96-well plates at a density of 1 × 10^4^ cells/mL and incubated at 37 °C with 5% CO_2_ for 24 h. This process was carefully repeated three times with cells from different passages to ensure experimental consistency and reliability.

The cytotoxicity of the test solutions was assessed using the MTT (3-(4,5-dimethylthiazol-2-yl)-2,5-diphenyltetrazolium bromide) assay, as described by Mosmann [54]. The analysis is grounded on the enzymatic reduction of the lightly colored tetrazolium salt to its purple formazan, which can be quantified spectrophotometrically where absorbance is directly proportional to the fraction of living cells. The absorbance was measured at 570 nm by a Synergy 2 multi-plate reader (BioTek Instruments, Inc., Highland Park, Winooski, VT, USA).

### 4.8. Assessment of Antioxidant Protection in an H_2_O_2_-Induced Oxidative Stress Model in HaCaT

The potential protective effects of test solutions—empty niosomes (N6), free CBD, single-loaded CBD (N:CBD), free EGCG, single-loaded EGCG (N:EGCG), and combination of free CBD and free EGCG (CBD + EGCG), combination of single-loaded CBD and single-loaded EGCG (N-CBD + N-EGCG), double loaded with CBD and EGCG (N/CBD + EGCG) and gel form (Gel/CBD+ EGCG) were evaluated using an in vitro model of H_2_O_2_-induced toxicity in HaCaT human keratinocytes. Cells were pre-treated with varying concentrations (0.1, 1 5, and 10 µM) of CBD and of 0.052, 0.52, 2.6, 5.2 µM of ECGC for 24 h, followed by exposure to H_2_O_2_ (200 µM) for 24 h. After the injury period, cell viability was evaluated by MTT assay.

#### Statistical Analysis

Statistical analysis was performed by GraphPad Prism 8 Software, version 8.0.2. The data were fitted to a one-way analysis of variance (ANOVA), followed by Dunnett’s multiple comparisons post-test to assess the differences between the control and treatment groups. A significance level of 0.05 was set as the threshold for determining statistical significance in all comparisons.

## Figures and Tables

**Table 1:** Composition and physicochemical characteristics of dual-loaded niosomes.

Niosome Formulations	Non-Ionic Surfactants/Chol (mol:mol)	CBD/Non-Ionic Surfactants (mol:mol)	D_h_ (nm) ± SD	PDI ± SD	ζ-Potential (mV) ± SD	EE (%) ± SD	
						CBD	EGCG
N1	Tw60:Sp60:Ch 3:3:4	1:10	177 ± 2.3	0.39 ± 0.05	−11.2 ± 1.8	78 ± 1.9	40 ± 0.9
N2	Sp60:Ch 6:4	1:10	186 ± 2.8	0.42 ± 0.04	−12.1 ± 2.4	80 ± 1.9	22 ± 1.1
N3	Tw60:Sp60:Ch 3.5:3.5:3	1:10	250 ± 1.8	0.44 ± 0.02	−10.9 ± 2.5	85 ± 0.9	50 ± 1.7
N4	Tw60:Sp60:Ch * 3.5:3.5:3	1:10	124 ± 5.5	0.36 ± 0.01	−13.8 ± 1.3	90 ± 1.4	-
N5	Tw60:Sp60:Ch ** 3.5:3.5:3	-	278 ± 4.3	0.32 ± 0.08	−12.6± 1.3	-	51 ± 0.6
N6	Sp60:Tw60:Ch *** 3.5:3.5:3	-	133 ± 4.8	0.34 ± 0.06	−12.3 ± 2.3	-	-

* CBD single-loaded niosomes; ** EGCG single-loaded vesicles; *** empty, non-loaded niosomes; Tw60, Tween 60; Sp60, Span 60; Ch, cholesterol; PDI, polydispersity index; EE, entrapment efficiency. Each value is presented as mean ± SD (n = 3).

**Table 2:** Physical stability evaluation of dual-loaded CBD/EGCG niosomes after storage at 4 °C for 30 days *.

Sample		Size (nm) ± SD	PDI ± SD	EE (%) ± SD	
				CBD	EGCG
Formulation N3	0 day	278 ± 4.3	0.32 ± 0.03	80 ± 0.9	50 ± 1.7
	30 days	280 ± 5.2	0.4 ± 0.05	76 ± 1.5	47 ± 2.6

* Each value is presented as mean ± SD (n = 3).

**Table 3:** Composition and gelling properties of plain ion-sensitive gels.

Formulation	Deionized Water (% *w*/*v*)	Gellan Gum (% *w*/*v*)	HPMC (% *w*/*v*)	Gelling Capacity	Gelation Time (s) ± SD
G1	99.9	0.1	-	-	-
G2	99.8	0.2	-	-	-
G3	99.7	0.3	-	+	92 ± 2.5
G4	99.6	0.4	-	++	80 ± 2.5
G5	99.5	0.5	-	+++	65 ± 2.5
G6	99.4	0.6	-	+++	40 ± 2.5
G7	99.55	0.2	0.25	+	90 ± 2.5
G8	99.25	0.2	0.5	++	88 ± 2.5
G9	99.45	0.3	0.25	+	90 ± 2.5
G10	99.2	0.3	0.5	++	60 ± 2.5
G6N*	47.6	0.6	-	+++	41 ± 1.3
G6N:CBD:EGCG**	47.6	0.6	-	+++	35 ± 2.1

G6N*—niosomal gel based on empty niosomes (see Table 1, formulation N6) and G6N:CBD:EGCG**—niosomal gel based on simultaneously loaded CBD; EGCG niosomes (formulation N3, Table 1). Each value is presented as mean ± SD (n = 3).

**Table 4:** Coefficient of determination (R^2^), release rate constants for each kinetic model (K_o_- zero order; K_1_ –first order; K_H_ – Higuchi and K_KP_ – Krosmeyer-Peppas), release half time (t_1/2_), and diffusion exponent (n), after fitting of release profiles to different drug release kinetic models.

Sample		Zero Order		First Order		Higuchi		Korsmeyer-Peppas			
	Kinetic Model	R^2^	K_o_ (mg/mL)/h	R^2^	K_1_ (h^−1^)	R^2^	K_H_ (mg/mL)/h^0.5^	R^2^	K_KP_ (h^−n^)	n	t_1/2_ (h)
N3:CBD		0.780	2.292	0.988	0.033	0.984	9.116	0.998	7.455	0.585	25.938
N3:EGCG		0.272	4.980	0.999	0.149	0.961	20.883	0.967	22.826	0.462	5.464
G6N:CBD		0.837	2.202	0.991	0.031	0.969	8.650	0.998	6.411	0.625	26.737
G6N:EGCG		0.437	4.586	0.995	0.130	0.956	19.279	0.961	21.181	0.460	6.482

**Table 5:** Micrographs of MRSA biofilm after exposure to blank (G6N) and hybrid niosomal G6N:CBD:EGCG gel (0.125/0.0625 mg/mL and lower).

Control	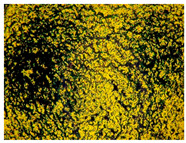	Blank	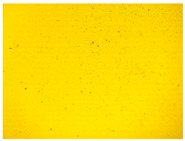
Samples	Dilution 1:8	Dilution 1:16	Dilution 1:32
G6N:CBD:EGCG	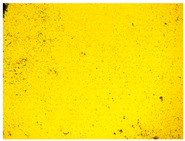	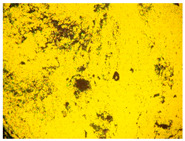	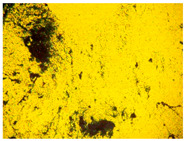
G6N	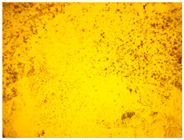	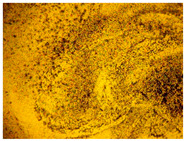	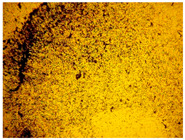

**Table 6:** Regression equation, retention time (t_R_) limit of detection (LOD), limit of quantification (LOQ) obtained for the compounds at 280 nm.

Analyte	t_R_ (min)	Regression Equation	R^2^	LOD (mg/mL)	LOQ (mg/mL)
EGCG	7.9	y = 51.33x + 2.2663	0.9987	0.01	0.003
CBD	13.7	y = 208.98x + 2.2926	0.9986	0.001	0.003

**Table 7:** Composition of the niosomal formulations.

Composition	Compo-sition Code	HLB Value	Surfactant: Cholesterol (Molar Ratio)	Total Lipid (µmol/mL)	CBD (µmol/mL)	EGCG (µmol/mL)
Tw60:Sp60:Ch	N1	9.85	3:3:4	30	3	4
Sp60:Ch	N2	4.7	6:4	30	3	4
Tw60:Sp60:Ch	N3	9.85	3.5:3.5:3	30	3	4
Tw60:Sp60:Ch	N4	9.85	3.5:3.5:3	30	3	-
Tw60:Sp60:Ch	N5	9.85	3.5:3.5:3	30	-	4
Sp60:Tw60:Ch	N6	9.85	3.5:3.5:3	30	-	-

Sp60—sorbitan monostearate; Tw60—polyoxyethylene (20) sorbitan monostearate; Ch—cholesterol.

## Data Availability

The original contributions presented in this study are included in the article/Supplementary Materials. Further inquiries can be directed to the corresponding author.

## Supplementary Materials

The following supporting information can be downloaded at: https://www.mdpi.com/article/10.3390/gels10120816/s1, Table S1: Statistical evaluation of biofilm formation—comparison between the different samples within one group (G6N or G6N:CBD:EGCG); Table S2: Statistical evaluation of biofilm formation—comparison between two groups (G6N vs. G6N:CBD:EGCG).
