# Supplementary material for: Development and Characterization of Dual-Loaded Niosomal Ion-Sensitive In Situ Gel for Ocular Delivery
Source: Gels. 2024 Dec 11;10(12):816. doi: 10.3390/gels10120816 (PMC11675977; doi:10.3390/gels10120816)
Supplement: Supplementary file 1 [file gels-10-00816-s001.zip › gels-3354280-supplementary.pdf]

Statistics

The statistical evaluation of the MRSA biofilm absorbance was performed using the using Two-way ANOVA (GraphPad Prism version 9.0.0 for Windows, GraphPad Software, Boston, Massachusetts USA). It is visible from the statistics results that the gel itself inhibits slightly the biofilm formation but there is no significant difference between the three dilutions of the gel. On the contrary, there is a significant difference ($p < 0.002$) between the dilutions of G6N:CBD:EGCG and the untreated control which reveals a concentration dependent biofilm inhibitory effect of the combination.

Table S1. Statistical evaluation of biofilm formation - comparison between the different samples within one group (G6N or G6N:CBD:EGCG).

Tukey's Multiple Comparisons Test	Mean Diff.	95.00% CI of Diff.	Below Threshold?	Summary	Adjusted *p* Value
**G6N**					
1:8 vs. Co	−0.3525	−0.4829 to −0.2221	Yes	***	0.0001
1:16 vs. Co	−0.2485	−0.3789 to −0.1181	Yes	**	0.0013
1:32 vs. Co	−0.2380	−0.3684 to −0.1076	Yes	**	0.0017
1:16 vs. 1:8	0.1040	−0.02639 to 0.2344	No	ns	0.1246
1:32 vs. 1:8	0.1145	−0.01589 to 0.2449	No	ns	0.0867
1:32 vs. 1:16	0.01050	−0.1199 to 0.1409	No	ns	0.9935
**G6N:CBD:EGCG**					
1:8 vs. Co	−0.7800	−0.9104 to −0.6496	Yes	****	<0.0001
1:16 vs. Co	−0.5280	−0.6584 to −0.3976	Yes	****	<0.0001
1:32 vs. Co	−0.2890	−0.4194 to −0.1586	Yes	***	0.0005
1:16 vs. 1:8	0.2520	0.1216 to 0.3824	Yes	**	0.0012
1:32 vs. 1:8	0.4910	0.3606 to 0.6214	Yes	****	<0.0001
1:32 vs. 1:16	0.2390	0.1086 to 0.3694	Yes	**	0.0017

Table S2. Statistical evaluation of biofilm formation - comparison between two groups (G6N vs. G6N:CBD:EGCG).

Šídák's Multiple Comparisons Test	Mean Diff.	95.00% CI of Diff.	Below Threshold?	Summary	Adjusted *p* Value
**G6N-G6N:CBD:EGCG**					
Co	0.000	−0.1300 to 0.1300	No	ns	>0.9999
1:8	0.4275	0.2975 to 0.5575	Yes	****	<0.0001
1:16	0.2795	0.1495 to 0.4095	Yes	***	0.0005
1:32	0.05100	−0.07902 to 0.1810	No	ns	0.6764
